# Accurate *in silico* confirmation of rare copy number variant calls from exome sequencing data using transfer learning

**DOI:** 10.1093/nar/gkac788

**Published:** 2022-09-16

**Authors:** Renjie Tan, Yufeng Shen

**Affiliations:** Department of Systems Biology, Columbia University, New York, NY 10032, USA; Department of Systems Biology, Columbia University, New York, NY 10032, USA; Department of Biomedical Informatics, Columbia University, New York, NY 10032, USA; JP Sulzberger Columbia Genome Center, Columbia University, New York, NY 10032, USA

## Abstract

Exome sequencing is widely used in genetic studies of human diseases and clinical genetic diagnosis. Accurate detection of copy number variants (CNVs) is important to fully utilize exome sequencing data. However, exome data are noisy. None of the existing methods alone can achieve both high precision and recall rate. A common practice is to perform heuristic filtration followed by manual inspection of read depth of putative CNVs. This approach does not scale in large studies. To address this issue, we developed a transfer learning method, CNV-espresso, for *in silico* confirming rare CNVs from exome sequencing data. CNV-espresso encodes candidate CNVs from exome data as images and uses pretrained convolutional neural network models to classify copy number states. We trained CNV-espresso using an offspring–parents trio exome sequencing dataset, with inherited CNVs as positives and CNVs with Mendelian errors as negatives. We evaluated the performance using additional samples that have both exome and whole-genome sequencing (WGS) data. Assuming the CNVs detected from WGS data as a proxy of ground truth, CNV-espresso significantly improves precision while keeping recall almost intact, especially for CNVs that span a small number of exons. CNV-espresso can effectively replace manual inspection of CNVs in large-scale exome sequencing studies.

## INTRODUCTION

Copy number variation refers to >50 bp deletion and duplication in the human genome (1,2). Copy number variants (CNVs), especially for rare CNVs, have been implicated in human diseases and phenotypic diversity (3–6). CNVs can be identified by many genomic technologies such as fluorescent *in situ* hybridization, array comparative genomic hybridization, single-nucleotide polymorphism array, next-generation sequencing (NGS) and long-read sequencing technologies (2,7–9). Among NGS technology, exome sequencing technology only performs sequencing on the coding regions that account for ∼2% of human sequence. Exome sequencing has the advantages of high efficiency, low cost and less storage spaces compared with whole-genome sequencing (WGS) technology. Therefore, exome sequencing is widely used as the main sequencing approach by numerous genomic studies of human diseases (10–12). Recently, many exome sequencing-based CNV detection methods have been developed (13–23). However, the accuracy of CNV detection from exome sequencing data is challenging, especially for small CNVs. The number of CNVs predicted by different methods varied from several to hundred CNVs per sample in which many of them are inconsistent calls (16,24,25).

To achieve a better performance, a common approach is to call CNVs from exome data using one or multiple methods, followed by empirical filtering based on summary metrics from these methods and then manual visualization of read pileups in regions that harbor candidate CNVs. This approach does not scale in studies with large sample size where manual inspection of a large number of candidate CNVs can be extremely time consuming (26–28). Furthermore, the quality of the manual inspection method is dependent on the experience of investigators, but even experienced investigators may make inconsistent judgments in different settings or times.

Here, we describe a new method, CNV-espresso, that can perform *in silico* confirmation of candidate rare CNVs by the same read depth summary figures used for manual visualization. The core model of CNV-espresso is a deep convolutional neural network (CNN). We represent a candidate CNV by an image showing the normalized read depth of the carrier sample and its corresponding reference samples. CNV-espresso uses CNNs pretrained by a large amount of image data (29) to perform transfer learning for predicting copy numbers as a classification question. In this study, we used a large-scale family-based exome sequencing dataset to create training data and evaluate the performance on samples with both exome sequencing and WGS data. We investigated whether CNV-espresso can improve accuracy beyond common empirical filters and how the improvement depends on the size and type of CNVs.

## MATERIALS AND METHODS

### Overview

**Figure 1.**
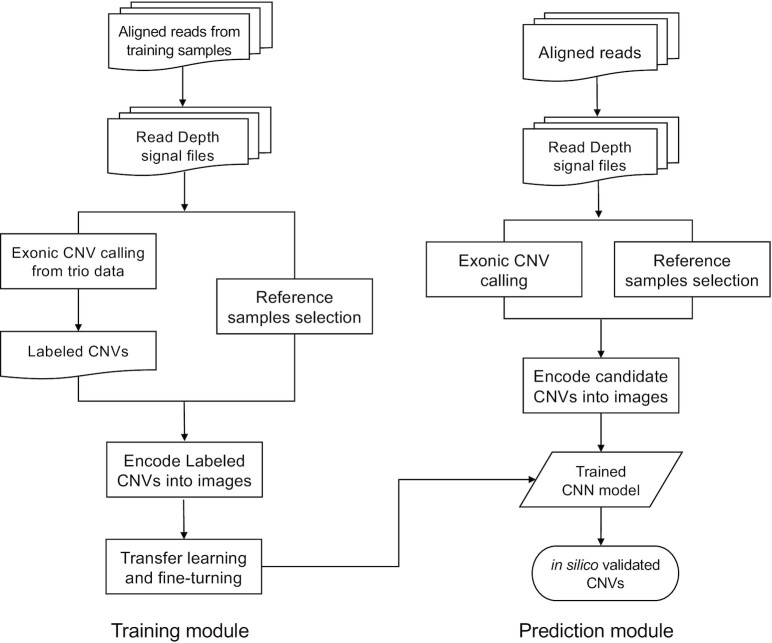
The workflow of CNV-espresso.

Figure 1 shows the overall workflow of this study. We construct training data by leveraging Mendelian inheritance of offspring–parents trio exome sequencing data. The core of CNV-espresso is a deep CNN model. We perform transfer learning on a pretrained image recognition model (29) using the constructed CNV, and then evaluate the performance of CNV-espresso on exome samples with WGS data as proxy of ground truth. We illustrate the overall workflow of CNV-espresso in Figure 1.

### Samples and CNV calling

We obtain exome sequencing data from 27 270 family-based samples in the autism spectrum disorder cohort (Simons Foundation Powering Autism Research for Knowledge, SPARK) (12). The samples are processed with custom NEB/Kapa reagents, the IDT xGen capture platform and sequenced on the Illumina NovaSeq 6000 system by Regeneron. Sequence reads are aligned to the human reference genome hg38.

We identify CNVs from exome sequencing data by using XHMM (13), CANOES (16) and CLAMMS (19), three CNV callers that have different statistical models. We use principal component analysis to illustrate samples and check whether the read depth signals of these samples have been significantly affected by batch effects. After passing the batch effect inspection, we randomly split samples into 10 groups (for XHMM and CANOES) or two groups (for CLAMMS) according to the computational complexity of different CNV callers. Note that in the random grouping process, we require members in the same trio to be assigned to the same group for CNV calling. In this study, we use all default parameters in CANOES and CLAMMS. For XHMM, we appropriately adjust the ‘--maxTargetSize’ parameter to keep 12 capture target probes >10 000 bp not being excluded as outliers. Except that, we keep all other parameters by default.

In addition to the 27 270 exome sequencing samples mentioned above, we also obtain additional 569 individuals with both exome sequencing and WGS data from SPARK (12). For these 569 individuals, we apply XHMM (13), CANOES (16), CLAMMS (19) and CODEX (30) to call putative CNVs from exome sequencing data. We limit the analysis to the rare CNVs (≤1% in the cohort) with two or more targets in the downstream analysis. Furthermore, we exclude CNVs with >75% of their intervals located in the segmental duplication regions. Here, segmental duplications refer to genomic duplications ≥1000 bp and ≥90% identity in the human reference genome (31). To get a compatible result from four different exome CNV callers, we further limit our analysis to autosomes. We use Canvas (32) and Manta (33) to identify CNVs from WGS data with default software parameters. We filter the raw CNV calls from Canvas and Manta by recommended quality control criteria. Specifically, we exclude CNVs with quality scores <7 in Canvas’ results; we only keep deletions and duplications located on autosomal and sex chromosomes (exclude any predicted variants on contigs) in Manta’s results. In addition, we require ‘PASS’ in the ‘FT’ field of the VCF file, which indicates that all the filters of Manta have passed for the corresponding sample.

### Training data

For these 27 270 exome sequencing samples from the SPARK cohort (12), we use XHMM (13), CANOES (16) and CLAMMS (19), three exome CNV callers that have different statistical models, to call candidate CNVs. To obtain training data without large-scale confirmation experiments, we use the Mendelian rule of inheritance to construct a high-confidence CNV call set. Specifically, in each family, we assume that most of CNVs called in both offspring and at least one parent that pass baseline quality filters are true positives and use these inherited calls as true rare deletions or duplications in the training of CNV-espresso. CNVs called only in offspring but not in either parent are Mendelian errors. These Mendelian error calls include false positives in offspring, false negatives in parents and true *de novo* CNVs. To refine the Mendelian error calls, we use baseline quality metrics to remove false negatives in parents. For instance, we require ‘${\rm NQ}$’ (a statistical quality score to reflect the Phred-scaled probability that none of the targets in this region have a CNV) (13) in both parents to be greater than a stringent threshold to achieve high probabilities of no CNVs in these regions. Meanwhile, based on recent studies, the real *de novo* CNV rate in the human genome is very low (34–36). To minimize the chance of uncertainty in the training data, we further exclude candidate CNVs identified by multiple callers, since we find that CNVs identified by multiple callers are likely to be real (Supplementary Figure S1). Then, we assume that most of the remaining Mendelian errors are mainly composed of false positives in offspring and we use these refined Mendelian error calls as *artifacts* for training. We list the detailed filtering approaches, quality scores and the thresholds used in this process in Supplementary Table S1. CNV-espresso is designed to confirm rare CNVs. Therefore, we exclude CNVs with a frequency over 1% in the cohort. As candidate CNVs came from three callers, we first group the overlapping candidate by CNV types and genomic coordinates, and then resolve the breakpoint conflicts by analyzing the read depth ratios between the inside of CNV regions and CNV boundary regions for all possible breakpoints. We process the merging steps by an external tool (25) and merge different sources of CNV calls into a unified call set.

### Read depth calculation and GC content normalization

To avoid the additional variance caused by the extremely long (≥1000 bp) exome capture targets, we first divide those extremely long targets into several equal size 500–1000 bp windows (referred to as ‘target’ in the manuscript). This approach can also benefit to confirm CNVs located at a part of the long target regions (19). We then employ Mosdepth (37) to calculate the read depth signal for each target of each sample. Users can also use other read depth generators to perform calculations or even directly take the read depth files from CLAMMS as input. It is well known that the real CNV signals in the exome sequencing data are significantly affected by noises. The noise signals include GC content, sample mean coverage, target size, target mean coverage, CNV frequency in the population, batch effects, etc. (13). These factors can affect read depth signal globally or locally. Among them, GC content and sample mean coverage are known to affect the read depth signal globally. Thus, we normalize the GC content and sample overall coverage for each sample by a median approach as

(1) $$\begin{equation*}{O_t} = \left( {{\rm RD}_t \times \frac{M}{{{M_x}}}} \right)/\bar M,\end{equation*}$$

where ${\rm RD}_t$ is the raw read depth at the $t$th exon. ${M_x}$ is the median read depth value of all exons with the same GC content $x$ as the exon and $M$ is the overall median read depth of all exons in a sample, while $\bar M$ is the overall mean read depth of all exons in a sample. ${O_t}$ is the normalized read depth value of the exon. Supplementary Figure S2 shows the read depth signal before and after GC content normalization.

### Reference sample selection

For other known and unknown factors locally affecting read depth signals, we assume that these factors contribute equally on the same batch of samples in the given genomic regions. A rare CNV in a case sample will lead to different normalized depth compared to other samples (as ‘reference’ samples) that have overall similar depth profile to the case sample. For instance, previous studies successfully detected rare CNVs by assuming the normalized number of read counts or read depth values in exome target regions follow a negative binomial or similar distributions (15,16). Inspired by these methods, we calculate the correlation coefficients of the case with other samples and select the 100 (by default) highest pairwise correlation samples as reference samples.

### Image encoding

For each CNV candidate predicted by CNV callers, we encode the read depth signals of the case and its corresponding reference samples into an image. The *X*-axis of the image refers to the CNV coordinate in the human genome and the *Y*-axis of the image refers to the normalized read depth value. To avoid image abnormalities caused by the fluctuations in distance changes between adjacent targets, we take the logarithmic transformation for the differences between two adjacent targets on the *X*-axis and the normalized read depth values of all exons on the *Y*-axis as equations (2) and (3), respectively:

(2) $$\begin{equation*}x_t^{\prime} = {x_0} + \sum\nolimits_{t = 1}^t {{{\log }_{\rm e}}\left( {1 + \left( {{x_t} - {x_{t - 1}}} \right)} \right)}, \end{equation*}$$

(3) $$\begin{equation*}y_t^{\prime} = {\log _{\rm e}}\left( {1 + {y_t}} \right),\end{equation*}$$

where ${x_t}$ is the genomic coordinate at the middle of $t$th exon and ${y_t}$ refers to the normalized read depth value at $t$th exon. To avoid the undefined error, we use the natural logarithm of one plus the input in the transformation.

In the image, each dot refers to a target region, and we connect every two adjacent dots of the sample with a straight line. We use two RGB colors to distinguish the case and reference of samples. Here, we use blue for the case sample and gray for reference samples and use Matplotlib (38) library in Python to implement the encoding process. Thus, we convert the CNV confirmation task into an image classification question.

### Transfer learning

In this study, we leverage the transfer learning and fine-tuning strategy to *in silico* confirm rare CNV calls from exome sequencing data. As requested by the pretrained base model, we resize our input CNV images to 224 × 224 pixels. Given that the purpose of our study is to train a three-class (rare deletion, rare duplication and artifacts) deep learning classifier, we exclude the top layer of MobileNet as the base model, and then add a global average pooling layer and a dense layer with Softmax activation function.

We apply the recommended transfer learning and fine-tuning procedures by Keras official developer guides (https://keras.io/guides/transfer_learning/). Specifically, we first take layers from the pretrained MobileNet base model and freeze all the weights as nontrainable to avoid destroying any of the information they already contained. Then, we train the weights of newly added layers using our labeled dataset. Once the model converges, we set all the weights of the model trainable and retrain the whole model end-to-end with a very low learning rate (10e−5) by Adam optimizer (39). We train the CNN model in batches of 32 images for up to 20 epochs on a GPU server (GeForce GTX 1080 GPU, 8119MiB RAM). Early stopping is set by monitoring the value of ‘loss’ and three epochs with no improvement after which training will be stopped. We select the model with the highest accuracy in the testing data as the final model.

### Evaluation metrics

We evaluate the performance of the method using withhold testing data and additional independent test datasets. Specifically, we use the refined model to predict the probabilities of the three copy number states (rare deletion, rare duplication and artifacts). We select the state corresponding to the maximum probability value as the predicted label. We treat a CNV as true positive if its predicated label matches the corresponding label. We count any true CNVs without a matched predicted label as false negatives, while any predicted CNVs without a matched true label as false positives. We evaluate the performance of CNV-espresso by using precision, recall, *F*_1_ score and area under the curve (AUC).

## RESULTS

### Model training

**Figure 2.**
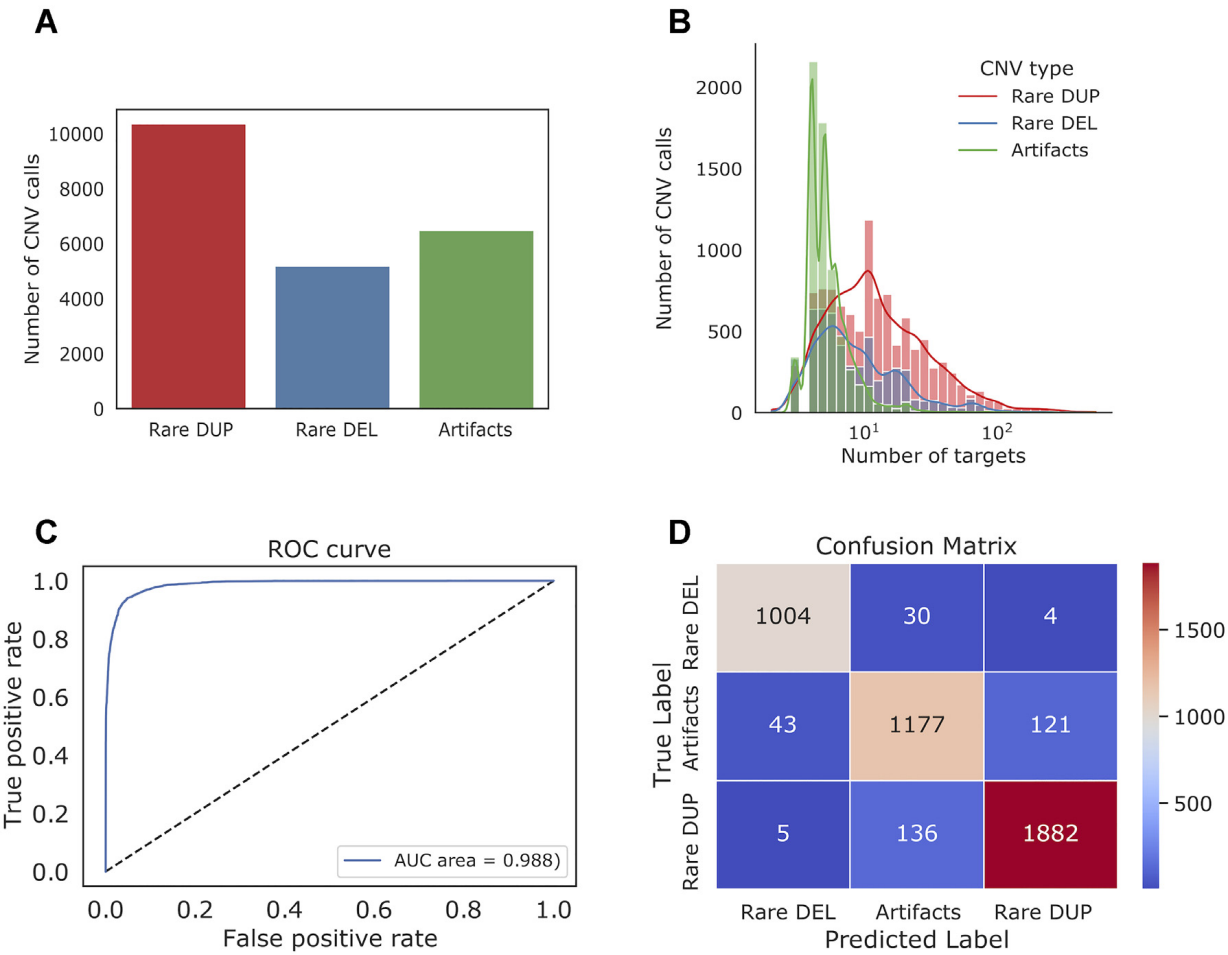
The distribution of CNVs and the model performance on the SPARK dataset. (**A**) The distribution of CNVs with different labels. (**B**) The distribution of labeled CNVs with different numbers of exome capture targets. Here, the number of targets can be used as the metric of CNV size. We shuffled the dataset and split CNVs into random training (60%), validation (20%) and testing (20%) data. (**C**) The receiver operating characteristic (ROC) curve of MobileNet after transfer learning and fine-tuning steps. The AUC of the ROC curve is added as a legend. (**D**) Confusion matrix. The true and predicted labels include rare duplication (Rare DUP), rare deletion (Rare DEL) and artifacts.

**Figure 3.**
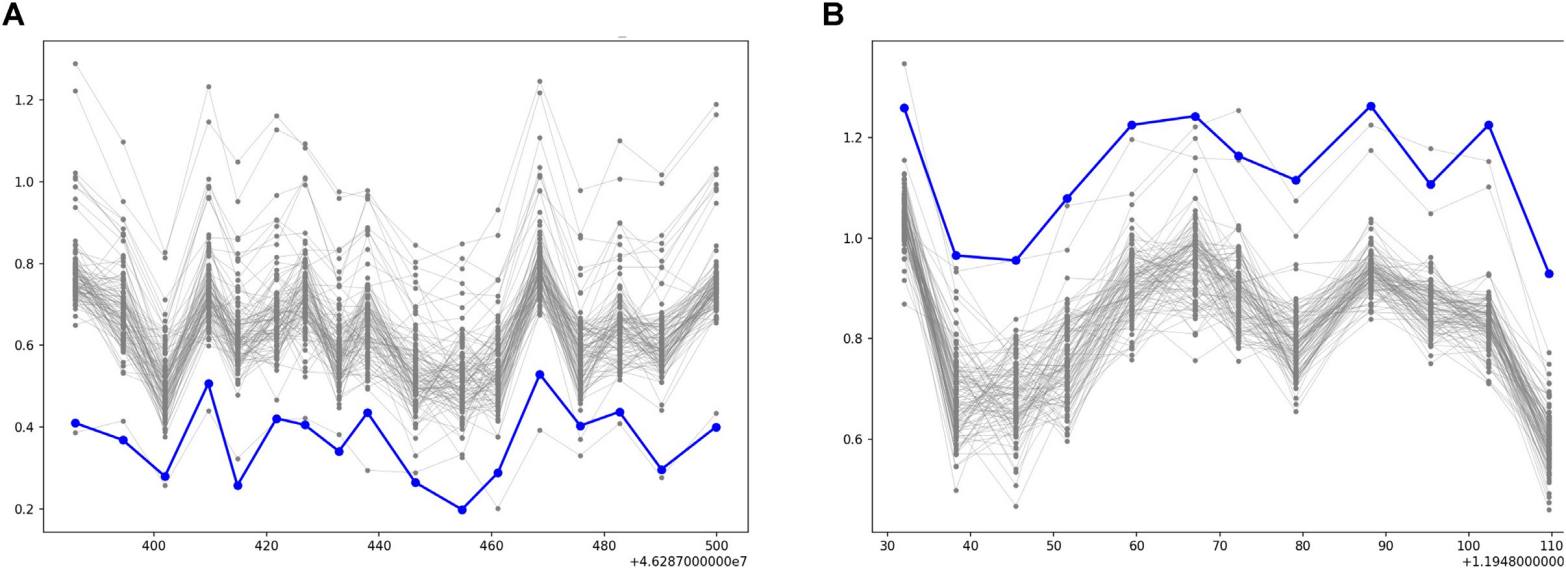
Representative images for CNV predictions. (**A**) Rare deletion. (**B**) Rare duplication. In the images, the case samples are encoded in blue (Hex code: #0000FF) and the reference samples are encoded in gray (Hex code: #808080).

We generated a dataset with 22 008 CNVs called from exome sequencing data of the SPARK project (see the ‘Materials and Methods’ section), including 10 354 rare duplications, 5180 rare deletions and 6474 likely artifacts (Figure 2A). Figure 2B shows the distribution of the size of CNV calls defined as the number of exome capture targets (referred to as ‘number of targets’). We randomly partitioned the data into training (60%), validation (20%) and testing (20%) data. Then, we encoded each CNV call as an image. Figure 3 shows the images of a rare deletion and a rare duplication as examples.

We leveraged the transfer learning approach, taking pretrained models learned from computer vision datasets on our exonic CNV confirmation task. We considered three pretrained CNN models: a generic CNN model, MobileNet v1 (29) and ResNet50 (40). The generic CNN model includes six convolutional layers, and it can achieve 0.84 accuracies on the CIFAR-10 dataset (41). MobileNet and ResNet have been successfully applied to genomics recently (26,42). Overall, all three models achieved acceptable classification performance. Among them, MobileNet achieved the highest performance with minimum parameters (Table 1). Therefore, we selected MobileNet as our transfer learning base model in the following analysis.

**Table 1.** Performance comparison and details of three CNN models

Model	Depth	# Parameters	Accuracy (95% CI)	*F*_1_ score (95% CI)	GPU time
A generic CNN	16	22 292 643	0.912 (0.90, 0.92)	0.912 (0.90, 0.92)	12
MobileNet v1	90	3 231 939	0.914 (0.90, 0.93)	0.915 (0.90, 0.93)	12
ResNet50	178	23 593 859	0.606 (0.39, 0.83)	0.603 (0.38, 0.82)	11

We calculated the mean (95% confidence interval, CI) accuracy, *F*_1_ score and GPU time in minutes in training data with 5-fold cross-validation.

After the transfer learning and fine-tuning steps (see the ‘Materials and Methods’ section), we evaluated the performance of the refined model in the testing data. The overall *F*_1_ score is 0.92, and the model achieves an AUC of the ROC curves at 0.99 (Figure 2C). The confusion matrix showed that our model can successfully classify most of the rare duplications, rare deletions and artifacts (Figure 2D).

To illustrate the relationship between CNV size and the performance of our model, we grouped the CNVs in the testing data into three categories according to the number of targets and evaluated our model on CNVs in each group. The performance is slightly better for large CNVs than small CNVs (Supplementary Figure S3). We also evaluated the model on deletions and duplications separately. Supplementary Figure S4 shows that the model achieved good performances on both deletions and duplications.

Although the training data are mildly imbalanced (Figure 2A), the learning process is well behaved (Supplementary Figure S5). To test whether balanced training data improve results, we used a downsampling approach to randomly select a subset of majority classes (rare duplication and artifacts) and construct balanced datasets. The model trained by the balanced set does not show improvement over the original model (Supplementary Figure S6).

### Performance of *in silico* confirmation of CNVs

**Figure 4.**
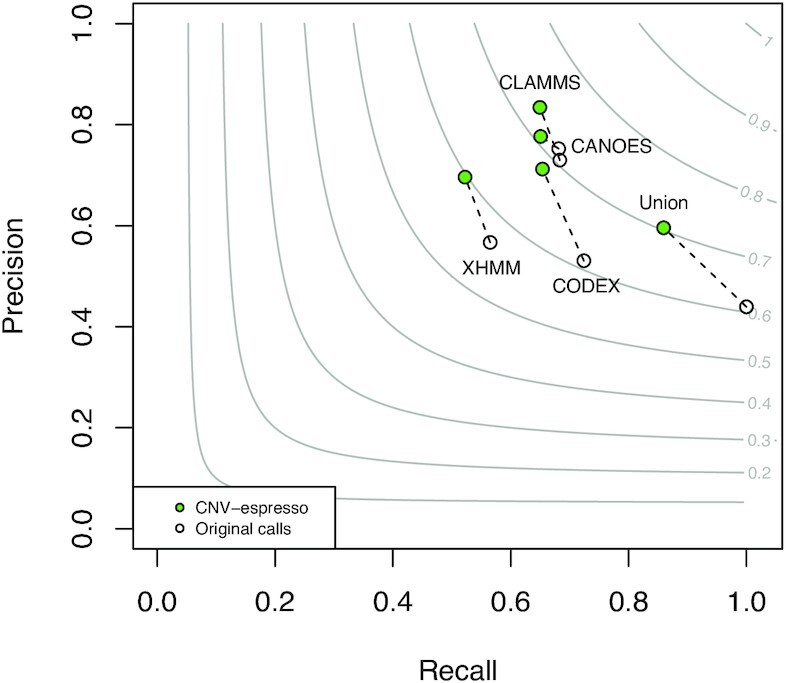
Overall performance comparison between original calls and *in silico* confirmation by CNV-espresso. XHMM, CANOES, CLAMMS and CODEX were used to call CNVs from exome sequencing data of 569 individuals. Canvas and Manta were used to call CNVs from WGS data of the same individuals. The union of all CNV calls from exome data that overlap with calls from WGS data was considered true positives. The calls from exome data that do not have support from WGS data were considered as false positives. The circles indicate CNV calls from corresponding CNV callers before (without color) and after (colored in green) *in silico* confirmation by CNV-espresso. The contour lines indicate the *F*_1_ score as the harmonic mean of the precision and recall.

WGS data have effectively complete coverage of genomic regions and more even coverage than exome sequencing data. Therefore, high-confidence CNVs identified from WGS data of the same individuals can be used as an approximate gold standard to assess the accuracy of CNVs called from exome data. In this study, we assessed the performance of CNV-espresso on 569 individuals with both exome sequencing and WGS data. For these 569 individuals, we used XHMM (13), CANOES (16), CLAMMS (19) and CODEX (30) to call putative CNVs from exome sequencing data, and then used Canvas (32) and Manta (33) to identify CNVs from WGS data (see the ‘Materials and Methods’ section). Given that Canvas and Manta are two complementary CNV callers in terms of different input signals and statistical models, we took the union of the high-confidence CNVs identified by the two callers as the standard dataset. We treated each CNV prediction from exome sequencing data as true or false based on whether at least 50% of the prediction in length overlaps with the corresponding one in the standard dataset. We estimated the precision of each method for exome data and the corresponding CNV-espresso filtered results as the true positives divided by all positives from the method. To estimate recall, we defined the total true positives as the union of CNV calls from exome data that have support from Canvas or Manta from WGS data. Figure 4 shows that CNV-espresso can improve the precision for all methods without incurring a substantial loss of recall. Consistently, CNV-espresso improved *F*_1_ score for all methods except CANOES. We note that CODEX was not used in constructing training data for the model. Supplementary Figure S7 showed that CNV-espresso can substantially exclude false-positive CNVs and improve the precision for calls made by CODEX.

We further investigated how CNV-espresso improves the results of XHMM when combined with its recommended quality score filtering approach. A commonly used method to filter XHMM calls is to set an ${\rm SQ}$ (genotype quality) threshold (a recommended value is 60). CNV-espresso improves the precision and overall performance of XHMM call sets with ${\rm SQ}$ thresholds at both 0 and 60 (Supplementary Figure S8). The improvement is more pronounced for small CNVs.

### Results on the experimentally validated dataset

To assess our model’s compatibility under different capture kits and experimental batches, we applied CNV-espresso to *in silico* confirm CNVs on a real experimentally validated dataset from a congenital heart disease study (43). We obtained 24 true positive CNVs validated by digital droplet polymerase chain reaction (ddPCR). The corresponding exome sequencing samples of these 24 CNVs were captured by Nimblegen SeqCap Exome V2 chemistry and sequenced on the Illumina HiSeq 2000 platform. Sequence reads were aligned to the human reference genome hg19 as described (43,44). We used our trained model to validate the CNV predictions for these 24 true CNVs. We found that CNV-espresso can successfully confirm 23 of the 24 CNVs previously picked up by exome sequencing technology with a true positive rate of 96% (Supplementary Table S2). We investigated the CNV call (a deletion) that was inconsistent with the experimental result. As shown in Supplementary Figure S9, the evidence from read depth supporting a true deletion in the offspring is weak. Additionally, the allele fraction data are inconsistent with a germline deletion. Therefore, it is challenging to confirm this deletion even by manual inspection.

## DISCUSSION

In this study, we present a new deep transfer learning method, CNV-espresso, for *in silico* confirming CNV predictions from exome sequencing data. In genomic studies using exome sequencing data, an indispensable step in CNV analysis is to manually visualize the images of putative CNVs that contain visual information about read depth. The core idea of CNV-espresso is to use deep learning models optimized for image recognition to capture implicit logics in manual visualization by humans. For each CNV candidate predicated by exome sequencing-based CNV callers, CNV-espresso encodes normalized read depth signal for the sample of interest and selected reference samples into an image. Then, CNV-espresso adopts transfer learning with a pretrained CNN model and fine-tuning the model by a large-scale trio exome dataset. We evaluate the performance of CNV-espresso on an independent dataset with both exome sequencing and WGS data and an experimentally validated dataset. Our results show that CNV-espresso can improve the precision for different exome sequencing-based CNV detection methods without incurring a substantial loss of recall. It can perform robustly on confirming both deletion and duplication. Furthermore, CNV-espresso can successfully *in silico* confirm CNV predictions among different size categories. Importantly, CNV-espresso can successfully confirm small CNVs with only a few targets, which is currently one of the biggest challenges in calling CNVs with exome area. Finally, our results also show that CNV-espresso can work compatibly with different capture kits and experimental batches and achieve a high true positive rate with ddPCR experimental results.

There are several limitations to our study. First, the main signal used by CNV-espresso is the depth contrast between the sample of interest and a selected set of reference samples. The presence of the same CNV in the reference samples would decrease the signal. Therefore, CNV-espresso is optimized for confirming rare CNVs. Second, CNV-espresso is designed to confirm CNV calls made by other methods. It can reduce false positives without substantial loss of sensitivity, and by design the model itself cannot improve sensitivity.

## DATA AVAILABILITY

The software is implemented in Python. The documentation and source code are freely available from the GitHub repository at https://github.com/ShenLab/CNV-Espresso.

The exome sequencing and WGS datasets from the SPARK project used in this work are available at https://base.sfari.org (application required).

## Supplementary Material

gkac788_Supplemental_File
