# Supplementary material for: Accurate in silico confirmation of rare copy number variant calls from exome sequencing data using transfer learning
Source: Nucleic Acids Res. 2022 Sep 16;50(21):e123. doi: 10.1093/nar/gkac788 (PMC9756945; doi:10.1093/nar/gkac788)
Supplement: gkac788_Supplemental_File [file gkac788_supplemental_file.pdf]

Supplementary figures and tables

Accurate *in silico* confirmation of rare copy number variant calls from exome sequencing data using transfer learning

Renjie Tan[1], Yufeng Shen[1, 2, 3]

1. Department of Systems Biology, Columbia University, New York, NY, USA
2. Department of Biomedical Informatics, Columbia University, New York, NY, USA
3. JP Sulzberger Columbia Genome Center, Columbia University, New York, NY, USA

Correspondence: R.T. (rt2776@cumc.columbia.edu) and Y.S. (ys2411@cumc.columbia.edu)

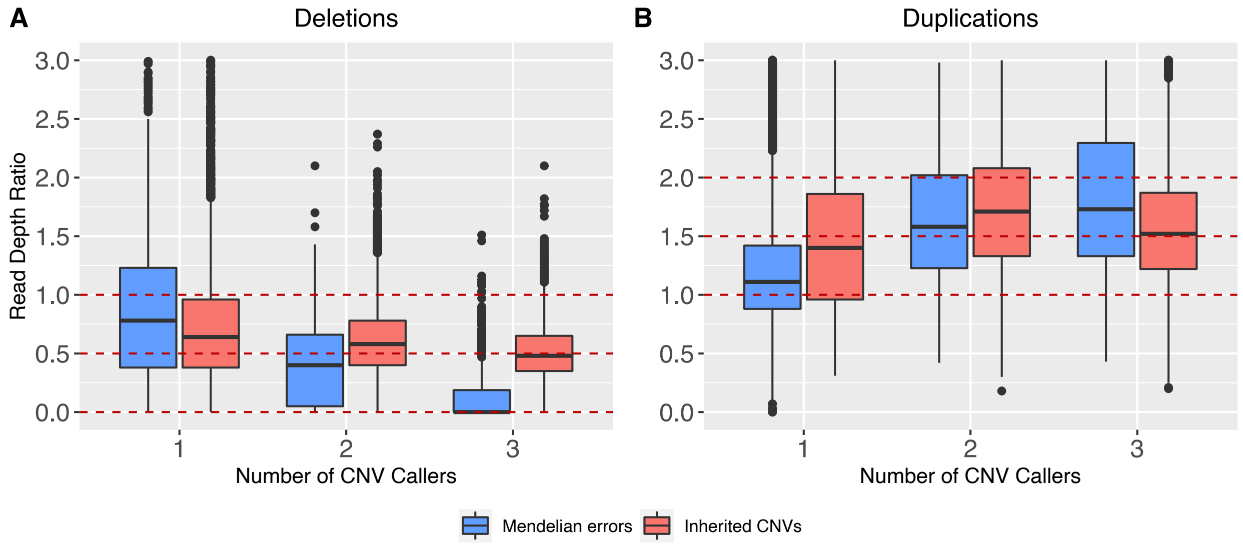

Supplementary Figure S1. Box plots illustrating the distribution of read depth ratio between CNV coordinates and their boundary regions versus different numbers of CNV callers in the labeled dataset. Deletion (A) and duplication (B) were illustrated separately and classified by inherited CNVs or Mendelian errors in different colors (Note that Mendelian error deletions and duplications were treated as *Artifacts* in the training data). For each CNV prediction, we first counted the number of exome targets located within the CNV coordinates, then selected half of this number of targets from each side of boundary regions. The read depth ratio was calculated as the average read depth within the CNV coordinates divided by the average read depth in left and right boundary regions. In theory, the read depth ratio should be close to 0 for homozygous deletions and 0.5 for heterozygous deletions; 1 for diploid regions, 1.5 and more for duplications. We observed that in the Mendelian error call set, as the deletions (duplications) were concordantly identified by more CNV callers, the read depth ratio has an obviously decreasing (increasing) trend. This means at least partly of CNV predictions concordantly identified by multiple CNV callers in the Mendelian error call set are likely to be real. However, precisely distinguishing the true and false *de novo* CNVs in the Mendelian error call set is challenging. Therefore, we treated those Mendelian error CNV calls identified by multiple callers as uncertainty, which need to be excluded from the final training data.

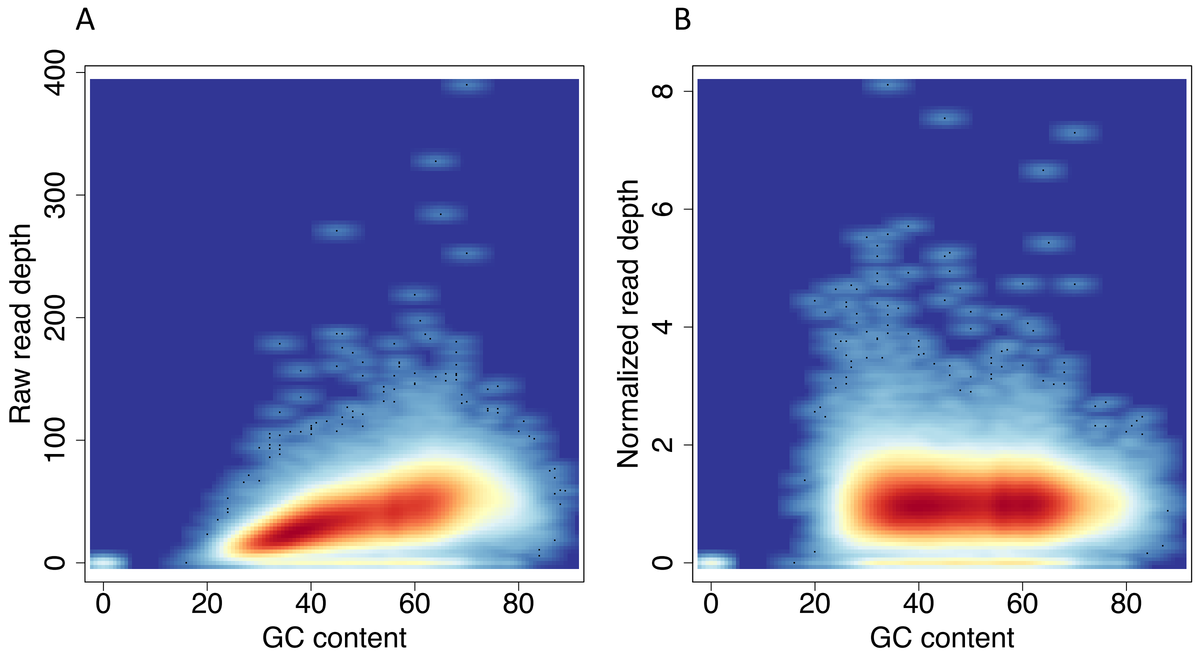

Supplementary Figure S2. GC normalization. (A) Correlation between GC content and the raw read depth before normalization. (B) Correlation between GC content and normalized read depth.

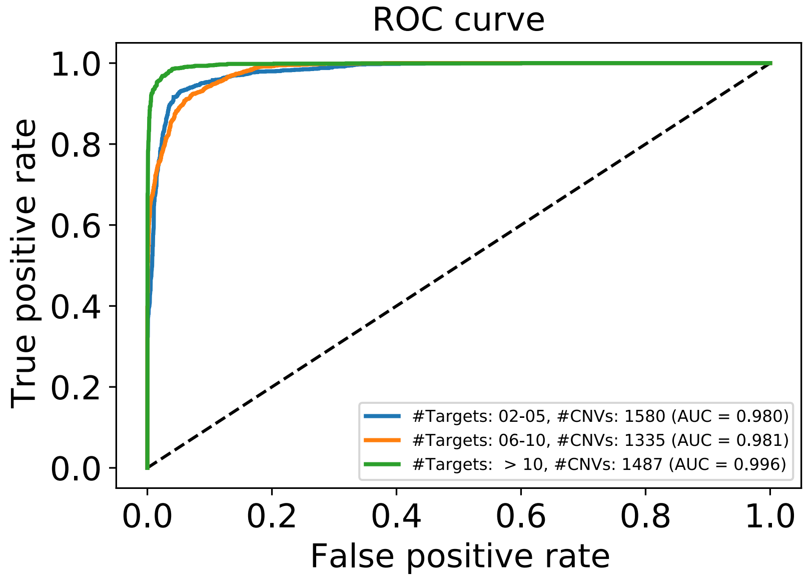

Supplementary Figure S3. Performance on different numbers of target intervals.

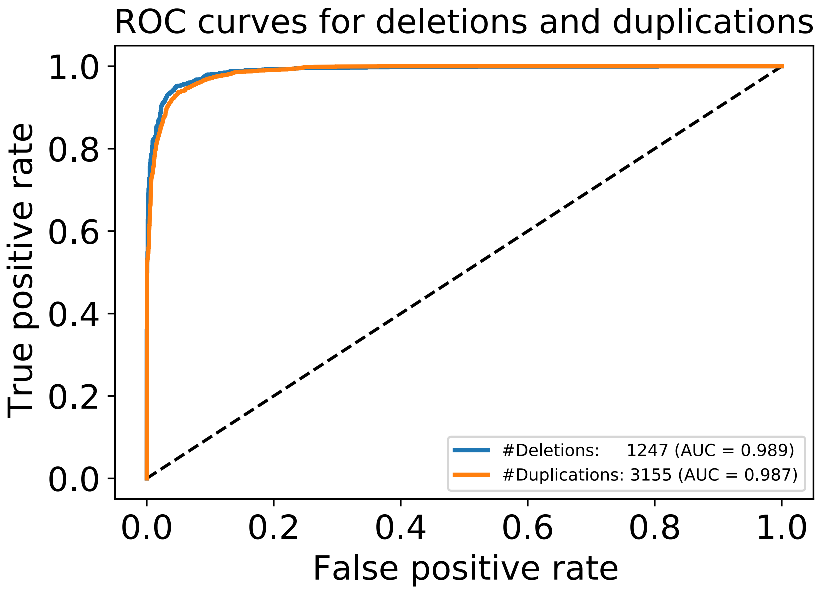

Supplementary Figure S4. Performance on deletions and duplications.

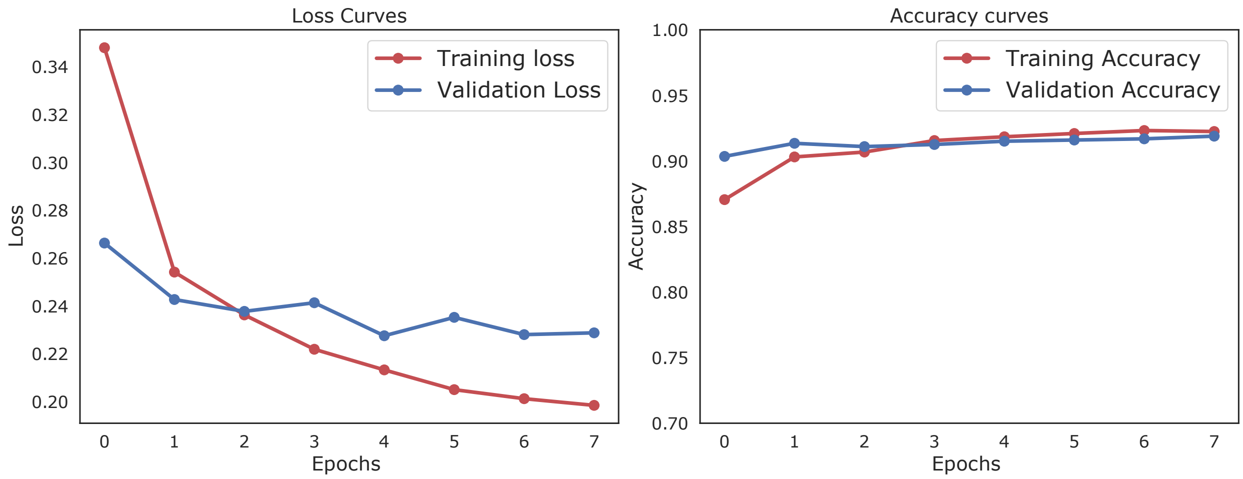

Supplementary Figure S5. Loss and accuracy curves in the transfer learning process.

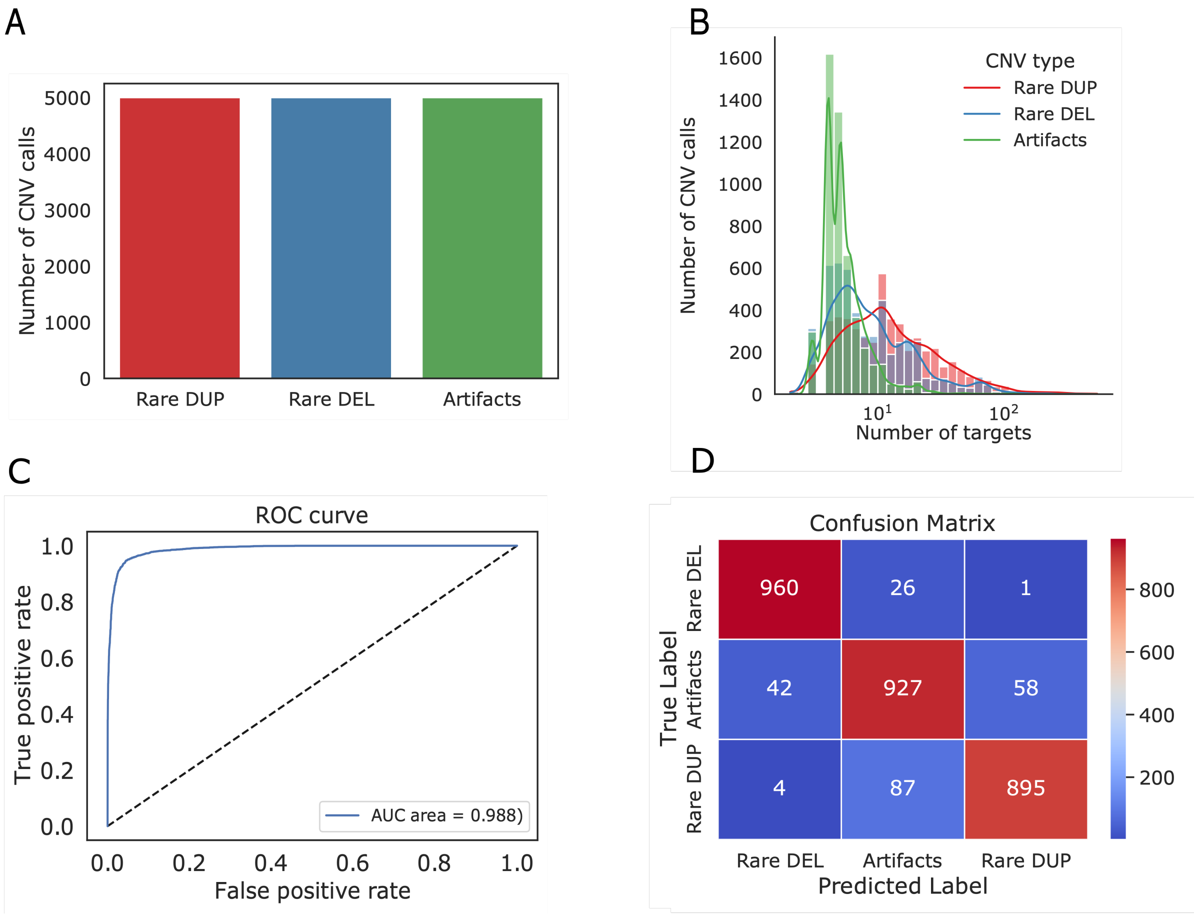

Supplementary Figure S6. Transfer learning on a balanced dataset.

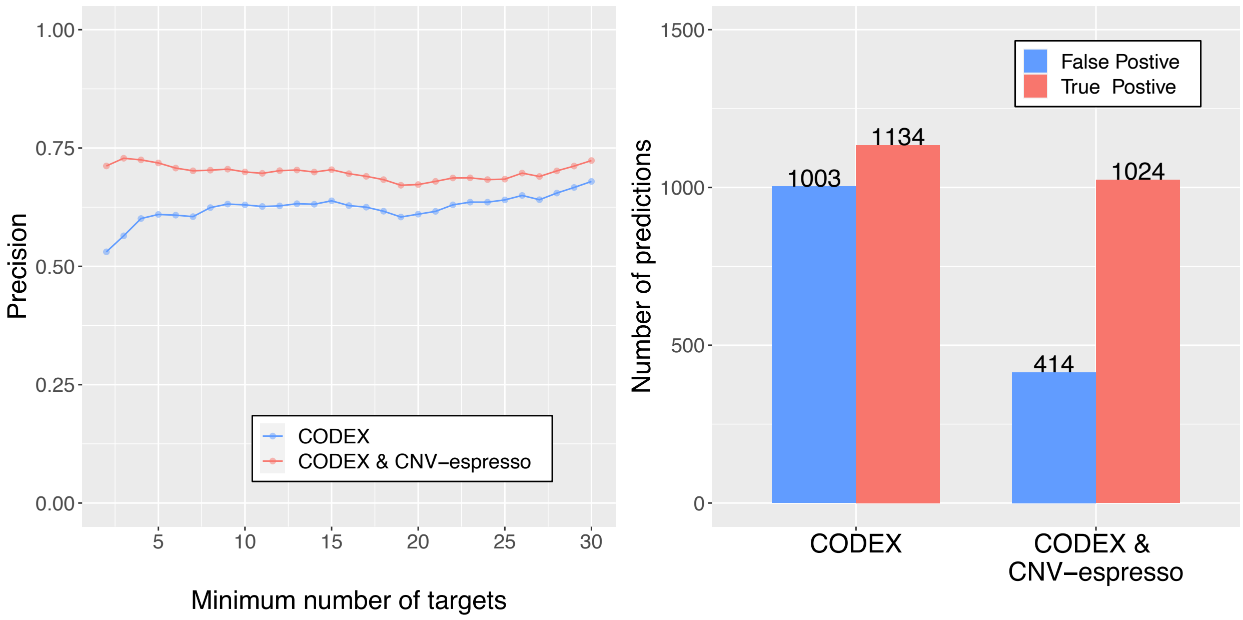

Supplementary Figure S7. Changes in precision and the number of CNVs predicted by CODEX before and after *in silico* confirmation by CNV-espresso. Target refers to exome capture target (Note that extremely long targets were split into equal size windows). To avoid the CNV calling quality issue, CNVs with a single target or more than 75% of their intervals located in the segmental duplication regions were excluded.

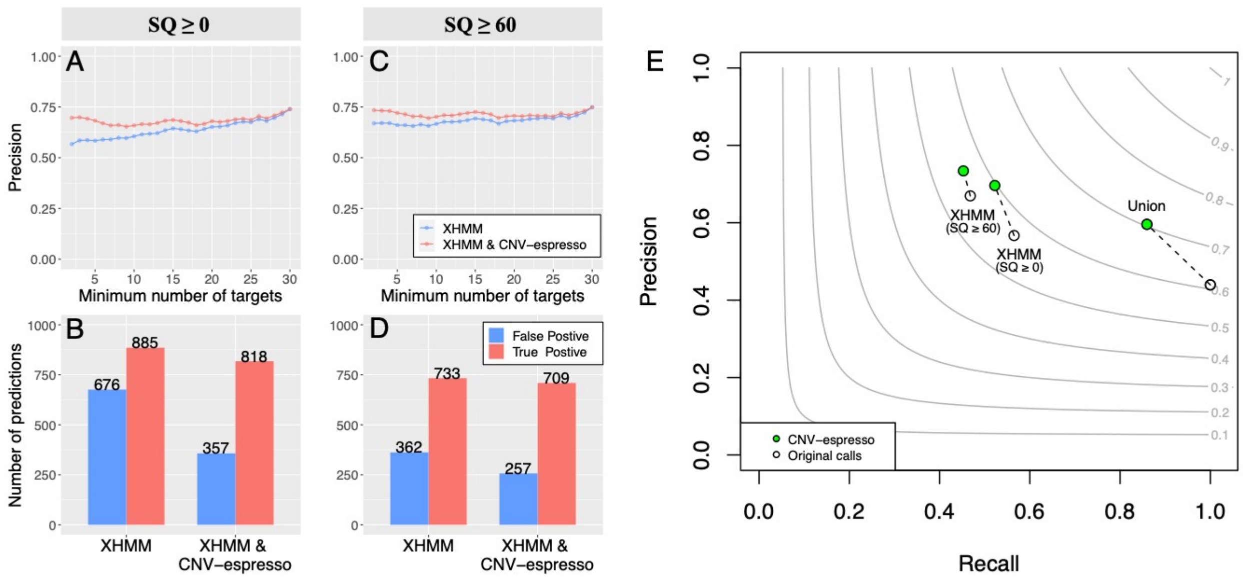

Supplementary Figure S8. Performance changes before and after *in silico* confirmation by CNV-espresso versus traditional quality score filtering approach. This analysis was conducted with different benchmark CNV call sets: (A, B) raw CNV predictions from XHMM. Note that all SQ scores of the raw CNV predictions are greater than 30 in our initial call set, thus there is no need to add '$SQ \geq 30$' category; (C, D) CNV predictions with SQ scores greater than or equal to 60, which is a recommended threshold for filtering in the XHMM pipeline. The plots in the lower panel were generated for all the classified CNVs with the corresponding SQ values marked at the top of this figure. For each benchmark call set, we checked the precision changes with different CNV sizes, as well as the number of true positive and false positive predictions before and after *in silico* confirmation by CNV-espresso. SQ was defined and estimated by XHMM. It refers to the relevant genotyping qualities as the Phred-scaled probability that any of the targets in that region have a CNV. Target refers to exome capture target (extremely long targets were split into equal size windows). To avoid the CNV calling quality issue, CNVs with a single target or more than 75% of their intervals located in the segmental duplication regions were excluded. (E) The precision and recall values showed the performance of XHMM call sets filtered with different SQ score thresholds and *in silico* confirmation by CNV-espresso. The precision-recall points of the union call set from four different exome CNV calling methods were given as a reference. The circles indicate CNV calls from corresponding CNV callers before (without color) and after (colored in green) *in silico* confirmation by CNV-espresso. The contour lines indicate the F1-score as the harmonic mean of the precision and recall.

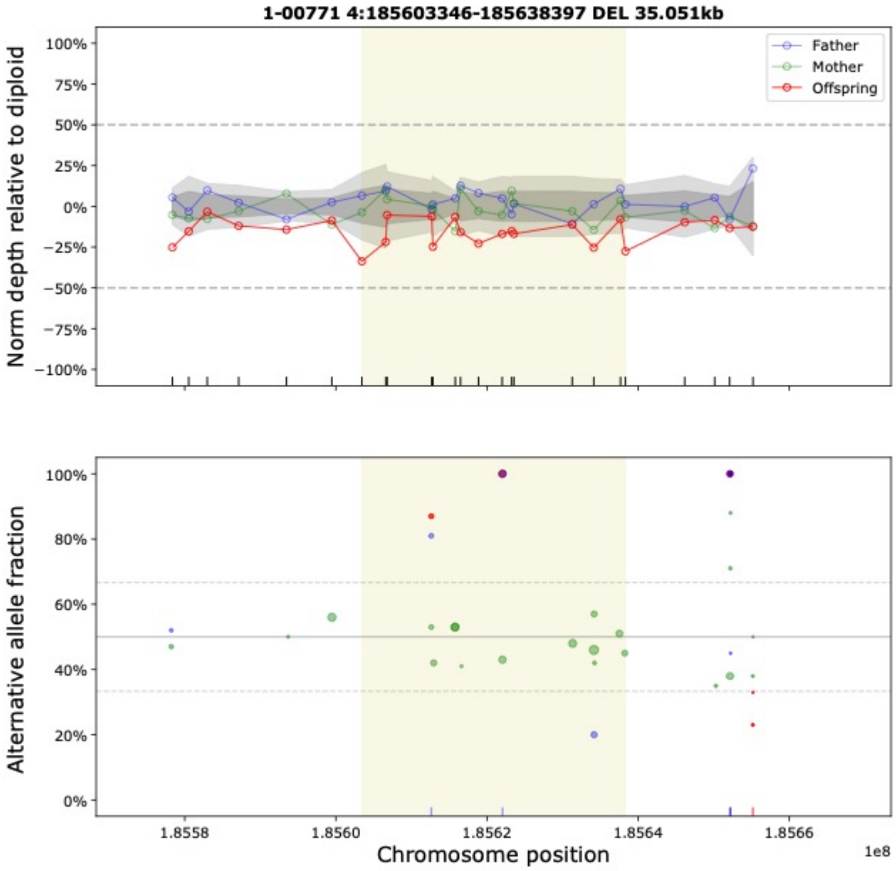

Supplementary Figure S9. Images were illustrated by read depth and alternative allele fraction signals for manual visualization. CNV regions were highlighted with a yellow background color.

Supplementary Table S1. Filtering criteria for generating training data using CNV calls made by three different methods

Training data	XHMM	CANOES	CLAMMS
Rare deletions and duplications (Inherited calls)	Offspring.SQ ≥ 60 & One.Parent.NQ ≥ 60 & Other.Parent.SQ ≥ 60	Offspring.SQ ≥ 70 & One.Parent.NQ ≥ 70 & Other.Parent.SQ ≥ 70	EQ > 0 & CNVs were shared (≥ 1 bp) by other CNVs in the parents
Artifacts (Mendelian errors)	Offspring.SQ ≥ 10 & Parents.NQ ≥ 60	Offspring.SQ ≥ 10 & Parents.NQ ≥ 70	EQ > 0 & SQ ≤ 80 & CNVs were NOT shared (≥ 1 bp) by any CNVs in the parents
Additional filtering criteria	1. Not located in segmental duplication regions, and<br>2. Not located in low Mappability (<0.25) regions, and<br>3. Not located in extreme GC content (<0.3 or >0.7) regions, and<br>4. Exclude Mendelian errors picked up two or three callers as uncertainty.		

SQ is the statistical quality score which refers to the Phred-scaled probability of some CNVs in the interval; NQ is the Phred-scaled probability of not being CNV in the interval, and EQ is the Phred-scaled probability of the exact CNV event in the given interval. These statistical quality scores could be estimated by the corresponding CNV calling tools.

Supplementary Table S2. *In silico* confirmation results on the experimentally validated dataset

ID	Ch	Start	End	Type	Size (kb)	Ref	#Targets	Prob_DEL	Prob_Artifact	Prob_DUP	Prob	Predicted_Label	Status
1-03171	1	145586403	145799634	DUP	213.2	hg19	50	0.000	0.007	0.992	0.992	DUP	Positive
1-01036	1	146631133	147416212	DUP	785.1	hg19	75	0.001	0.005	0.994	0.994	DUP	Positive
1-01401	2	102493466	103001458	DEL	508	hg19	52	1.000	0.000	0.000	1.000	DEL	Positive
1-01401	2	145155868	145274931	DEL	119.1	hg19	9	0.588	0.411	0.002	0.588	DEL	Positive
1-01049	3	15637812	15643461	DUP	5.6	hg19	4	0.000	0.014	0.986	0.986	DUP	Positive
1-00771	4	185603346	185638397	DEL	35.1	hg19	15	0.044	0.956	0.000	0.956	Artifacts	Negative
1-01049	6	43484783	43485159	DUP	0.4	hg19	2	0.000	0.004	0.996	0.996	DUP	Positive
1-00096	7	50179707	50191153	DEL	11.4	hg19	3	1.000	0.000	0.000	1.000	DEL	Positive
1-00977	7	138258252	143807632	DEL	5549.4	hg19	674	0.510	0.004	0.486	0.510	DEL	Positive
1-01995	7	142334207	142460871	DEL	126.7	hg19	17	0.999	0.001	0.000	0.999	DEL	Positive
1-00566	8	11606428	11710963	DEL	104.5	hg19	26	1.000	0.000	0.000	1.000	DEL	Positive
1-00230	11	86939592	87025456	DEL	85.9	hg19	6	1.000	0.000	0.000	1.000	DEL	Positive
1-01486	11	125641368	134943190	DEL	9301.8	hg19	473	1.000	0.000	0.000	1.000	DEL	Positive
1-01049	14	74551632	74551731	DUP	0.1	hg19	1	0.032	0.456	0.512	0.512	DUP	Positive
1-01396	15	22750305	23228712	DEL	478.4	hg19	63	1.000	0.000	0.000	1.000	DEL	Positive
1-00243	15	22835893	23062345	DEL	226.5	hg19	61	1.000	0.000	0.000	1.000	DEL	Positive
1-01994	15	28389771	28446734	DEL	57	hg19	25	1.000	0.000	0.000	1.000	DEL	Positive
1-01696	15	44833588	44856873	DEL	23.3	hg19	8	1.000	0.000	0.000	1.000	DEL	Positive
1-01995	17	38544624	38548586	DEL	4	hg19	6	0.999	0.001	0.000	0.999	DEL	Positive
1-01049	17	39845210	39846477	DUP	1.3	hg19	3	0.000	0.005	0.995	0.995	DUP	Positive
1-00113	22	18886915	22000000	DEL	3113.1	hg19	535	1.000	0.000	0.000	1.000	DEL	Positive
1-01836	22	19020529	21380382	DEL	2359.9	hg19	453	1.000	0.000	0.000	1.000	DEL	Positive
1-00425	22	36038076	36149338	DEL	111.3	hg19	9	1.000	0.000	0.000	1.000	DEL	Positive
1-00197	X	148685645	148693146	DUP	7.5	hg19	3	0.000	0.000	1.000	1.000	DUP	Positive

These 24 experimentally validated CNVs were obtained from Glessner et al., 2014. The probabilities of rare deletion (Prob_DEL), artifact (Prob_Artifact), and rare duplication (Prob_DUP) for each CNV were predicated by CNV-espresso. The CNV type corresponding to the maximum probability value (Prob) was selected as the predicted label. A 'Positive' *in silico* confirmation result was given if the predicated label matches the corresponding label (Type), otherwise 'Negative' was given.
